# Error detection for radiotherapy planning validation based on deep learning networks

**DOI:** 10.1002/acm2.14372

**Published:** 2024-05-06

**Authors:** Shupeng Liu, Jianhui Ma, Fan Tang, Yuqi Liang, Yanning Li, Zihao Li, Tingting Wang, Meijuan Zhou

**Affiliations:** ^1^ Department of Radiation Medicine Guangdong Provincial Key Laboratory of Tropical Disease Research, NMPA Key Laboratory for Safety Evaluation of Cosmetics School of Public Health Southern Medical University Guangzhou Guangdong China; ^2^ Department of Radiation Oncology Nanfang Hospital Southern Medical University Guangzhou Guangdong China; ^3^ Department of Clinical Engineer Nanfang Hospital Southern Medical University Guangzhou Guangdong China

**Keywords:** CNN multi‐classification model, error detection, GPR method, three‐dimensional dose validation

## Abstract

**Background:**

Quality assurance (QA) of patient‐specific treatment plans for intensity‐modulated radiation therapy (IMRT) and volumetric modulated arc therapy (VMAT) necessitates prior validation. However, the standard methodology exhibits deficiencies and lacks sensitivity in the analysis of positional dose distribution data, leading to difficulties in accurately identifying reasons for plan verification failure. This issue complicates and impedes the efficiency of QA tasks.

**Purpose:**

The primary aim of this research is to utilize deep learning algorithms for the extraction of 3D dose distribution maps and the creation of a predictive model for error classification across multiple machine models, treatment methodologies, and tumor locations.

**Method:**

We devised five categories of validation plans (normal, gantry error, collimator error, couch error, and dose error), conforming to tolerance limits of different accuracy levels and employing 3D dose distribution data from a sample of 94 tumor patients. A CNN model was then constructed to predict the diverse error types, with predictions compared against the gamma pass rate (GPR) standard employing distinct thresholds (3%, 3 mm; 3%, 2 mm; 2%, 2 mm) to evaluate the model's performance. Furthermore, we appraised the model's robustness by assessing its functionality across diverse accelerators.

**Results:**

The accuracy, precision, recall, and F1 scores of CNN model performance were 0.907, 0.925, 0.907, and 0.908, respectively. Meanwhile, the performance on another device is 0.900, 0.918, 0.900, and 0.898. In addition, compared to the GPR method, the CNN model achieved better results in predicting different types of errors.

**Conclusion:**

When juxtaposed with the GPR methodology, the CNN model exhibits superior predictive capability for classification in the validation of the radiation therapy plan on different devices. By using this model, the plan validation failures can be detected more rapidly and efficiently, minimizing the time required for QA tasks and serving as a valuable adjunct to overcome the constraints of the GPR method.

## 1 INTRODUCTION

Intensity‐modulated radiation therapy (IMRT) and volumetric modulated arc therapy (VMAT) have emerged as the most prevalent radiotherapy techniques globally.^1^ Their prime advantages include potent dose modulation capacity, highly conformal target dose, and sharp dose gradients on tumor targets. However, they necessitate stringent accuracy in dose delivery and linear accelerator performance for quality assurance (QA) tasks.^2^, ^3^

To ensure precise dose delivery in radiation therapy, it is incumbent upon clinical practice to conduct patient‐specific QA for each patient's radiation therapy plan before initiation of treatment. The American Association of Physicists in Medicine (AAPM) and the Radiation Therapy Oncology Group (RTOG) have developed a series of guidelines and standards for linear accelerators, treatment planning systems (TPS), and radiation therapy plans, to evaluate whether related tasks comply with clinical standards.^4^, ^5^, ^6^, ^7^

In order to accomplish a comprehensive evaluation tool capable of assessing dose plateau region, dose gradient region, and low dose region in IMRT plans, the conventional gamma pass rate (GPR) method is typically employed to appraise each measurement point in the plan.^8^ When the pass rate fails to meet the threshold standard, plan validation is deemed unsuccessful and the causes for this verification failure need to be pinpointed. The plan then must be revised, re‐measured, and re‐evaluated. This procedure can be lengthy and burdensome in clinical work, primarily because determining the reasons for failure can be challenging. A plan can only be considered validated once the pass rate reaches the threshold, after which patients can continue treatment.^9^

The GPR method could benefit from enhancement and supplementation. Previous research has suggested that GPR outcomes do not reliably correlate with specific errors that manifest during actual irradiation.^10^, ^11^ A study conducted by Carlone et al.^12^ leveraged receiver operating characteristic (ROC) analysis to assess GPR performance, and it was found to be relatively insensitive to minor errors in multi‐leaf collimators (MLC). Moreover, the exclusive use of GPR analysis does not adequately delineate the causes of plan verification failures. This lack of specificity makes it challenging to identify appropriate adjustments for radiation therapy plans, thereby complicating the process of revising these plans.^13^, ^14^

The remarkable advancements in deep learning within the field of artificial intelligence have stimulated a growing body of research exploring its application for QA research in radiation therapy. Some studies have utilized deep learning methodologies to predict the GPR of radiotherapy plans by incorporating complexity metrics, fluence maps, and dose distribution as input data, thereby forecasting the points of failure in GPR.^15^, ^16^, ^17^, ^18^

Other research efforts have concentrated on formulating models to predict error types in MLC specifically for patient‐specific IMRT QA, using the radiomics features acquired by Electronic Portal Imaging Devices (EPID) dosimetry.^19^ Furthermore, several studies have engineered machine learning‐based dose analysis methods for IMRT QA. For instance, Wootton et al. developed a machine learning model for error detection in patient‐specific IMRT QA using radiomic analysis of QA images.^20^ Nyflot et al. proposed an error classification model that merges prior methods with CNN models.^21^ Similarly, Kimura et al. simulated patient‐specific VMAT QA MLC positional errors utilizing a cylindrical ionization chamber array and constructed a CNN model for classifying MLC error types from measured and predicted dose distributions.^22^, ^23^

However, the practical application of most research findings in clinical settings remains challenging and there exists ample scope for improvement. The concept of predicting GPR is promising, as it can streamline the repetitive plan validation process. However, without actual measurements, the GPR obtained from the predictive model cannot uncover instances where the radiation therapy plan fails due to hardware errors in linear accelerators during execution. In reality, most of the failures of the plan are attributed to mechanical and dose errors that occur during irradiation. Furthermore, the GPR method presents issues in error analysis as it evaluates results solely based on a single indicator, thereby lacking sensitivity to error types and their causes.^24^, ^25^, ^26^ For instance, the prediction of MLC positional errors based on EPID measurement solely detects error types caused by MLC hardware. However, in clinical practice, this type of error is rarely encountered. Moreover, error prediction utilizing EPID and 2D plans dosimetric verification devices is incapable of accurately reflecting 3D level errors due to the parallel alignment of the accelerator gantry and the measurement plane.

In general, hardware‐related errors such as gantry errors, collimator errors, couch errors, and dose errors are inherent in accelerator equipment. Various international guidelines and standards^4^, ^5^, ^6^, ^7^ have specified tolerance intervals for these errors. However, some studies have not based their error scenarios and error types on this tolerance range, leading to the detection of errors that lack sufficient clinical significance. Moreover, most model validations are confined to a single device or technique (IMRT or VMAT), and cross‐validation across different devices and techniques is absent. This may suggest that the model's robustness is deficient and necessitates extensive data collection and training in diverse environments.

Existing research continues to grapple with the issues mentioned above. As such, this study, operating within the tolerance limits of the linear accelerator, designs distinct error plans for both IMRT and VMAT techniques, measures the dose maps of these plans utilizing a cylindrical three‐dimensional dose validation device, and then incorporates these data into a CNN model. The goal of this study is to juxtapose the deep learning method with the GPR method and delve into the advantages offered by the deep learning approach in the quality control of plan validation. The results garnered from this study can serve as a valuable supplement to the GPR method and facilitate its application in clinical practice.

## 2 METHODS

### 2.1 Treatment plans and measurement devices

This retrospective study analyzed the IMRT and VMAT plans that had been verified and treated at our hospital. A total of 94 patients were used for model training and testing, with a total of 300 radiation therapy plans, including 198 IMRT plans and 102 VMAT plans. The treatment sites and radiotherapy techniques of the patients are presented in Table 1. The Varian Trilogy linear accelerator (Varian Medical Systems, Palo Alto, CA, USA) was used for the administration of the radiation therapy plan, with the photon energy of 6 MV and Millennium 120MLC. The Eclipse 15.1 TPS was used to create the plans with 2.5 mm grid spacing for QA dose calculation using the Acuros External Beam (AXB) version. Furthermore, the Varian VitalBeam linear accelerator was used to verify the model's robustness with the Eclipse TPS. A total of 15 verification patients, including 30 IMRT plans and 20 VMAT plans, were randomly selected.

**TABLE 1:** Distribution of tumor treatment sites and treatment techniques.

Tumor site	Numbers	IMRT	VMAT
Brain	10	10	0
Head & Neck	16	4	12
Breast	24	23	1
Abdomen	34	13	21
Others	10	10	0
Total	94	60	34

Abbreviations: IMRT, intensity‐modulated radiation therapy; VMAT, volumetric modulated arc therapy.

### 2.2 Error types selection and conventional evaluation methods

Based on the AAPM 198 QA guidelines on linear accelerator error tolerance requirements,^6^ this study divided plan errors into five categories (normal plans, gantry errors, collimator errors, couch errors, and dose errors) to ensure that the training and validation of this study can be achieved on different equipment and techniques with the same tolerance as the clinical guidelines. Based on AAPM 119 and AAPM 218 reports,^5^, ^7^ the GPR method was evaluated using conventional evaluation methods.^8^

### 2.3 Data collection and processing

**FIGURE 1:**
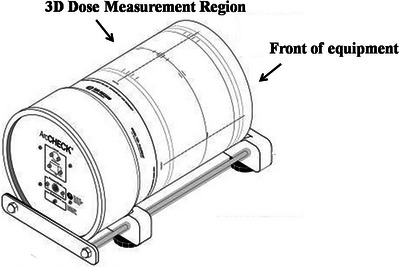
Structure of ArcCHECK cylindrical phantom for 3D dose validation equipment.

The calculated plans were created in the TPS based on different types of errors, including gantry rotation error of ± 1°, collimator rotation error of ± 1°, couch rotation error of ± 1°, and dose error of ± 2%. As it was not possible to create gantry rotation errors in VMAT plans, an additional 34 IMRT patient plans were selected to supplement the training data and balance the dataset of 5 classes. A total of 300 plans were generated for the 94 patients, with 60 plans per class. Since the main cause of plan validation failures is generally one of the error types, the data collection for this study included only one error type. The linear accelerator and the plan verification device underwent general QA quality control calibration prior to measurement, including mechanical accuracy, accelerator output, ArcCHECK measurement array, and ArcCHECK dose to ensure that the accuracy of the machine meets the requirements. For error plans, we develop them in TPS and ensure that each error plan contains only one error. Meanwhile, we use error‐free plans for actual measurements. The GPR results are generated by comparing the error plans from the TPS with the error‐free plans from the actual measurements. The calculated plan files were exported as DICOM format and generated the dose fluence into the ArcCHECK (as shown in Figure 1), recording the passing rates under different GPR standards. The dose files of the TPS and ArcCHECK devices have different dose grid and resolution. We re‐sampled the data to match the same data dimension as the TPS data and the measurement data. Due to the significant difference in prescription dose among different patient plans, all data were normalized to improve the model's convergence speed and accuracy.

Lastly, a single training dataset encompasses both measurement data and calculated data from one patient. A total of 300 sets of such data were utilized as input data, and the five classes of error types were randomly partitioned into training and testing sets using a 3:1 ratio. During the model training phase, data augmentation was carried out on the training data to expand the dataset to 1125 sets. Furthermore, to evaluate the model's robustness, 50 plans sourced from VitalBeam accelerators were chosen for the verification dataset (Table 2).

**TABLE 2:** Number of training, testing, and validation dataset in the study.

	Normal plan	Gantry error	Collimator error	Couch error	Dose error
Training dataset	225	225	225	225	225
Testing dataset	15	15	15	15	15
Verification dataset	10	10	10	10	10

*Note*: The dataset comprising 300 instances was partitioned into training and testing sets in a ratio of 3:1. The model training was conducted utilizing the 5‐fold cross‐validation methodology. Subsequently, the model's performance was assessed on a test dataset of 75 cases. An external validation of the model's performance was also conducted using a verification dataset of 50 cases, which were acquired from a different linear accelerator.

### 2.4 Construction of CNN model

**FIGURE 2:**
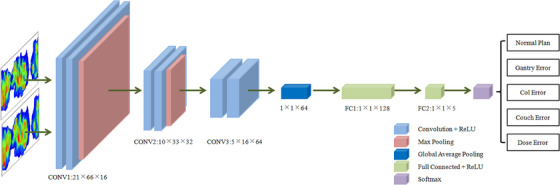
Schematic diagram of CNN network model.

The general structure and dimensional changes of the classification prediction model for error detection are shown in Figure 2 and Table 3, respectively. The model is a multi‐classification based on CNN model that can detect each type of error separately. The input of this study is based on the dual‐channel input of the dose fluence based on TPS and measurement devices, and the output is a 5‐classification task. The Adaptive Moment Estimation (ADAM)algorithm is used to optimize loss and update network parameters, and NVIDIA GPU RTX3090 graphics card is used for model training. All code writing work was done in the Pytorch framework.

**TABLE 3:** CNN model configuration and data dimensions.

Layer type	Configuration	Input	Output
Input		(2,21,66)	(16,21,66)
Convolution 1	3 × 3 Kernel	(16,21,66)	(16,21,66)
BatchNorm1		(16,21,66)	(16,21,66)
ReLU1		(16,21,66)	(16,21,66)
Max pooling 1	2 × 2 Pooling	(16,21,66)	(16,10,33)
Convolution 2	3 × 3 Kernel	(16,10,33)	(32,10,33)
BatchNorm2		(32,10,33)	(32,10,33)
ReLU 2		(32,10,33)	(32,10,33)
Max pooling 2	2 × 2 Pooling	(32,10,33)	(32,5,16)
Convolution 3	3 × 3 Kernel	(32,5,16)	(64,5,16)
BatchNorm3		(64,5,16)	(64,5,16)
ReLU3		(64,5,16)	(64,5,16)
Global average pooling		(64,5,16)	(64)
Full Connected 1		(64)	(128)
Dropout	0.5 Dropout	(128)	(128)
Full Connected 2		(128)	(5)
Softmax		(5)	(5)

### 2.5 Model training and testing

**FIGURE 3:**
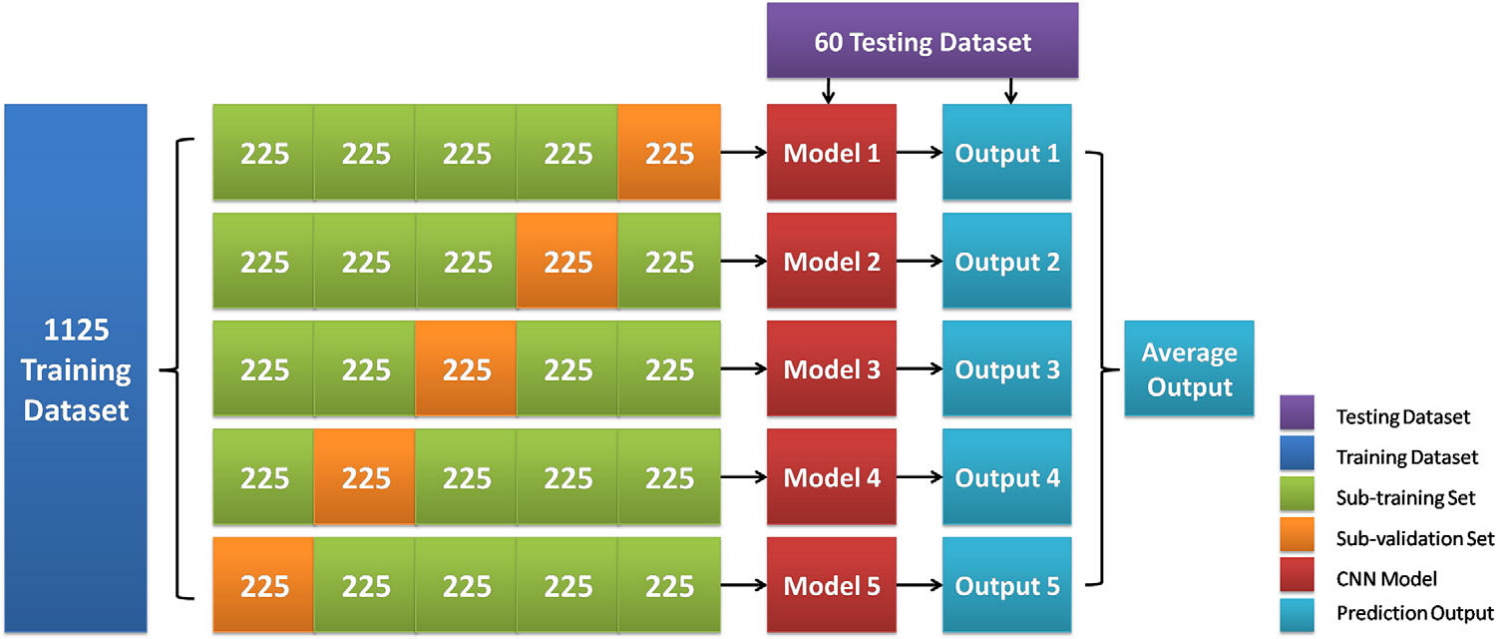
Flowchart for 5‐fold cross validation.

To avoid over‐fitting of the prediction model, we adopted the 5‐fold cross‐validation^26^, ^27^ method for training. First, we divide the 1125 training dataset into five portions of 225 data each. Second, four of them are taken each time for model training, and the remaining one is used as the cross‐validation set. After that, the training is repeated five times to get five cross‐validation models. The predicted value of the overall model is obtained by averaging the results of the five models. Finally, the testing dataset is added to the model for prediction to evaluate the performance of the model. The process of cross‐validation is shown in Figure 3.

In our study, all labels are in binary and each bit represents an error type, for example, (1, 0, 0, 0, 0) represents type 0 and (0, 1, 0, 0, 0) represents type 1. Since the training dataset in this study contains only one type, we use the Softmax function as the output prediction, and the probability values of the five type predictions are summed up to one. In addition, the model uses a hyper‐parameter search method for optimal parameter selection, the hyper‐parameters include epoch = [300, 400, 500], batch size = [16, 32, 64, 128], learning rate = [0.0001, 0.0005, 0.001, 0.005]. With different parameter choices, the performance of the model is compared using the testing dataset to find out the best performance of the model. The optimized parameters epoch = 500, batch size = 16, learning rate = 0.0005 are finally obtained.

### 2.6 Evaluation standards for the classification prediction model and GPR

The evaluation standards for conventional GPR were set up as three types: (1) (3%, 3 mm, 10% threshold), the GPR pass rate was greater than 95% for plan approval. (2) (3%, 2 mm, 10% threshold), the GPR pass rate is greater than 90% for plan approval. (3) (2%, 2 mm, 10% threshold). Because there was no clear pass rate requirement for the third standard in the current guidelines, we adopted a relatively low pass rate in this study. GPR greater than 85% was considered pass. For the evaluation of the performance of the CNN model, we used classic indicators such as the ROC curve,^28^ AUC, accuracy, recall, precision, and F‐value to evaluate the model and compared the results with those obtained under GPR. The calculation method of GPR combines the dose error ratio with the distance‐to‐agreement (DTA) error, as shown in the formula below:

(1) $$\Gamma \left(\bar{r}m,\bar{r}c\right)=\sqrt{\frac{\gamma^{2}\left(\bar{r}m,\bar{r}c\right)}{\Delta d_{M}^{2}}+\frac{\delta^{2}\left(D_{m}\left(\bar{r}m\right),D_{c}\left(\bar{r}c\right)\right)}{\Delta D_{M}^{2}}}$$

(2) $$r\left(\bar{r}m,\bar{r}c\right)=\left|\bar{r}m-\bar{r}c\right|$$

(3) $$\delta \left(D_{m}\left(\bar{r}m\right),D_{c}\left(\bar{r}c\right)\right)=D_{m}\left(\bar{r}m\right)-D_{c}\left(\bar{r}c\right)$$

(4) $$\gamma \left(\bar{r}m\right)=\min\left\{\Gamma \left(\bar{r}m,\bar{r}c\right)\right\}\forall \left\{\bar{r}m\right\}$$

where $\bar{r}m$ and $\bar{r}c$ are the vector positions of the evaluated and reference points, respectively, $D_{m}\left(\bar{r}m\right)$ and $D_{c}\left(\bar{r}c\right)$ are the evaluated and reference doses, respectively, $\Gamma (\bar{r}m,\bar{r}c)$ is the numerical value of GPR at a measurement point. $r(\bar{r}m,\bar{r}c)$ represents the distance difference between the measurement points and the TPS calculation points. $\delta (\bar{r}m,\bar{r}c)$ is the dose difference (DD) between the measurement points and the TPS calculation points. $\Delta d_{M}^{2}$ and $\Delta D_{M}^{2}$ are the DTA and dose evaluation thresholds under different GPR standards. When $\gamma \bar{r}m\leq 1$, it is considered that the measurement point passes the dose verification. GPR is the percentage of gamma ≤1 in all analyzed points with D ≥ threshold. The calculation formulas for evaluation indicators of prediction model performance such as accuracy, recall, precision, and F1 score are shown below:

(5) $$\mathrm{Accuracy}=\frac{\mathrm{TP}+\mathrm{TN}}{\mathrm{TP}+\mathrm{TN}+\mathrm{FP}+\mathrm{FN}}$$

(6) $$\mathrm{Recall}=\frac{\mathrm{TP}}{\mathrm{TP}+\mathrm{FN}}$$

(7) $$\mathrm{Precision}=\frac{\mathrm{TP}}{\mathrm{TP}+\mathrm{FP}}$$

(8) $$F1\;\mathrm{score}=\frac{2*\mathrm{Precision}*\mathrm{Recall}}{\mathrm{Precision}+\mathrm{Recall}}$$

TP represents true positive results, TN represents true negative results, FP represents false positive results, and FN represents false negative results.

## 3 RESULTS

### 3.1 Quality control workflow for the validation of the radiation plan

**FIGURE 4:**
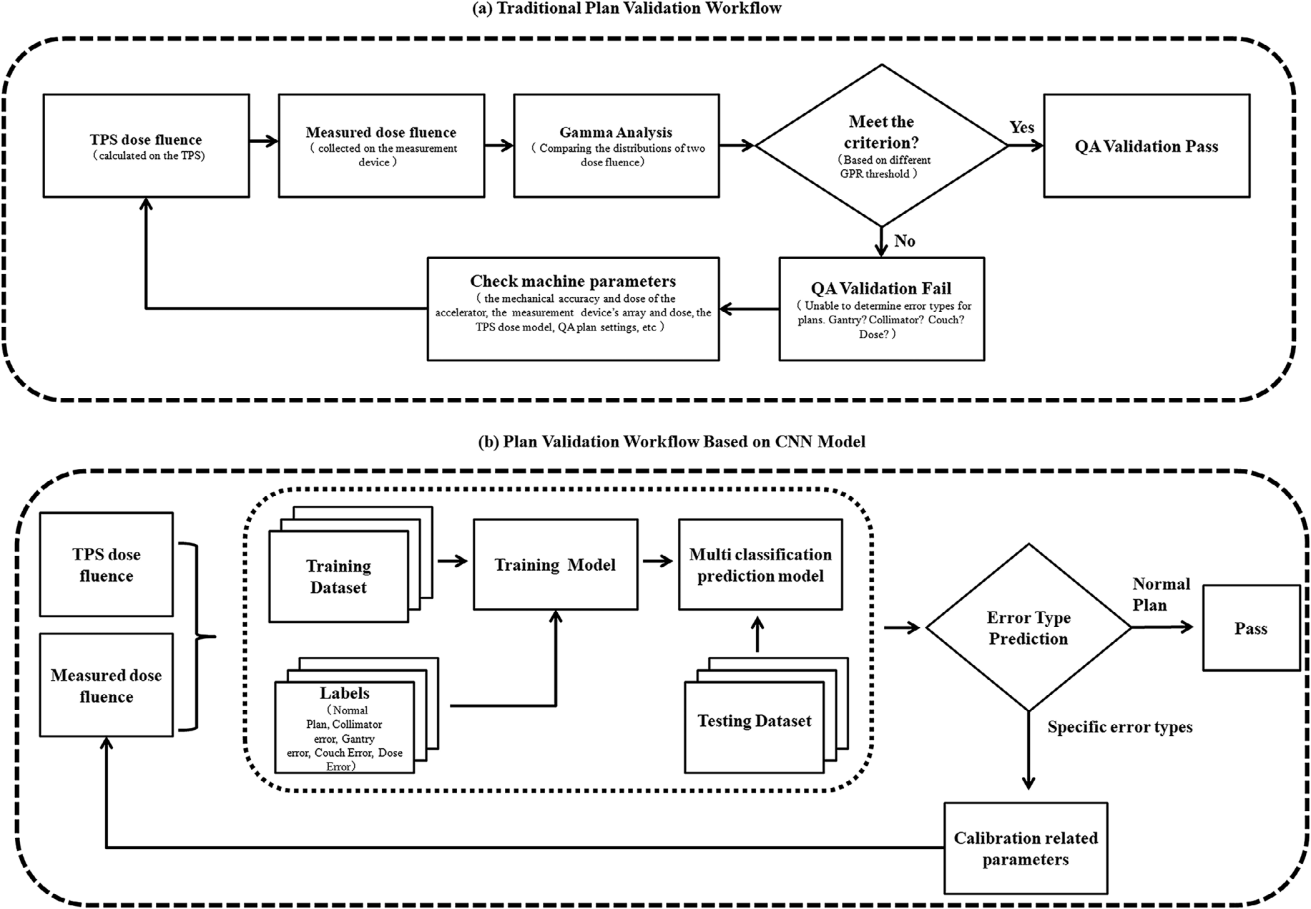
Workflows for two different plan validation methods.

The QA quality control workflow for radiotherapy patients expected to be achieved in this study is shown in Figure 4, along with the conventional quality control procedures. To improve the efficiency of the QC process and create new QC analysis methods and solutions, we constructed a classification prediction model based on deep learning networks.

### 3.2 Comparison between prediction models and the GPR method

**FIGURE 5:**
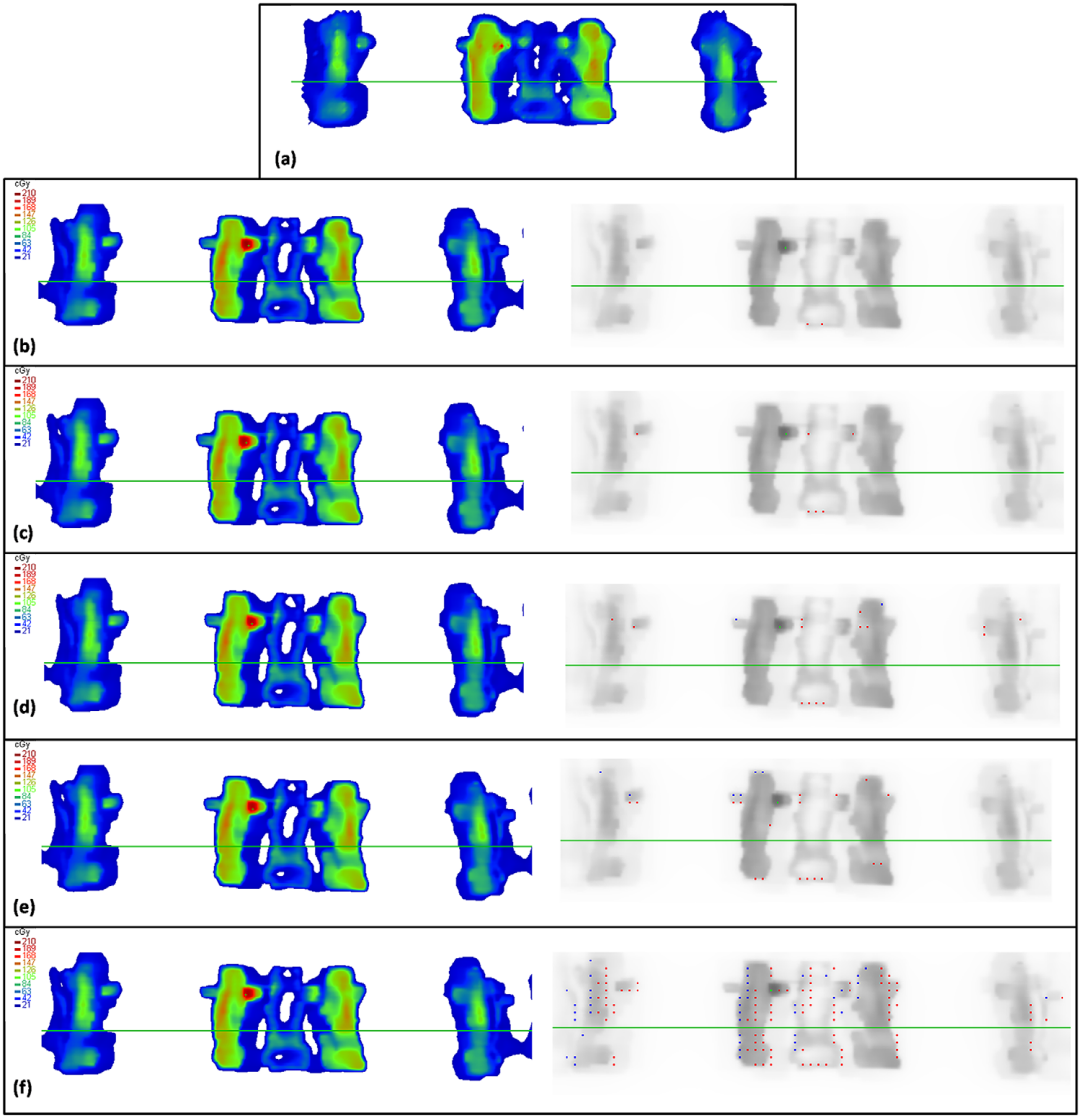
Comparison of the measured dose fluence and TPS‐calculated dose fluence for different types, as well as the distribution of GPR pass points. Red points represent the failure points higher than the measured dose under the GPR threshold, while blue points represent the failure points lower than the measured dose. (a) Measurement dose fluence, (b) normal plan, (c) dose error, (d) collimator error, (e) couch error, and (f) gantry error.

**FIGURE 6:**
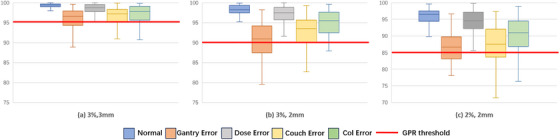
Box plots of normal plans and error types under different gamma pass rate (GPR) thresholds.

The dose fluence maps and distribution of gamma pass/fail points for different error types are shown in Figure 5. The GPR method can only obtain the specific numerical value of the plan pass rate but cannot directly distinguish the error type. Therefore, we calculated the numerical distribution under different GPR thresholds. Figure 6 show that the GPR method has extremely limited judgment ability for all errors under different threshold values. These results indicated that the current GPR method cannot accurately evaluate plans with distinct types of error and cannot reflect situations where the equipment and dose tolerance limits are exceeded during the implementation of the radiation therapy plan.

**FIGURE 7:**
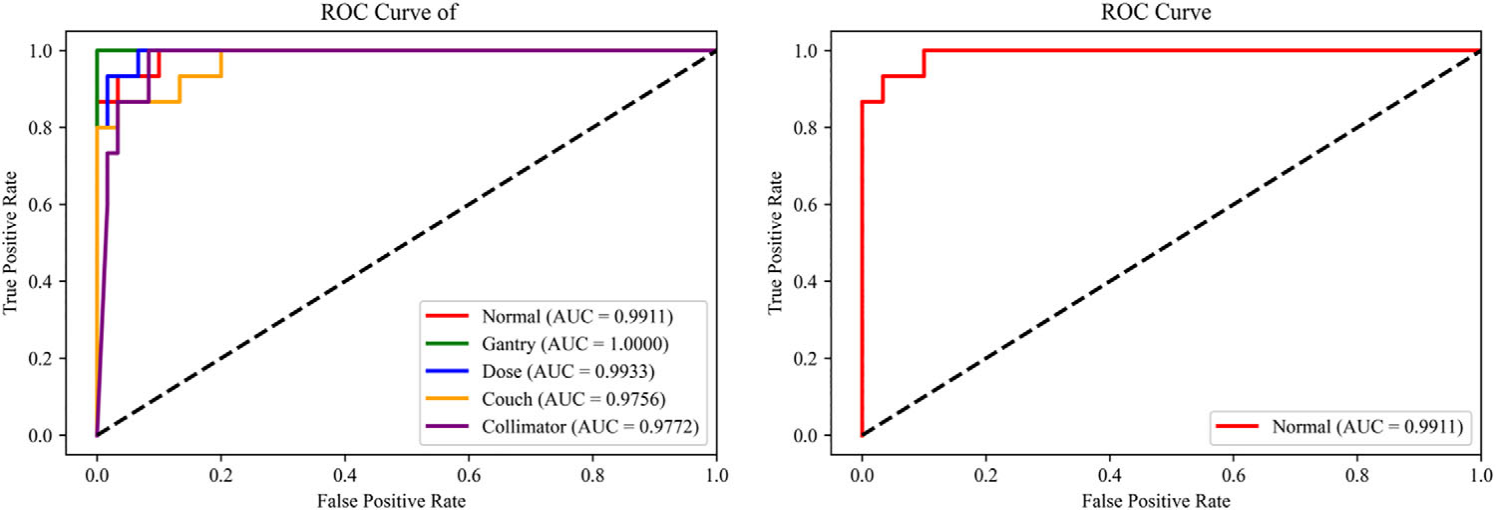
The receiver operating characteristic (ROC) curve of the multi‐classification model and normal plan versus error plan.

In the current study, the relevant data were incorporated into the model for training and testing, and the corresponding ROC curve was generated. As depicted in Figure 7, the area under the curve (AUC) values were considerably high for all error types. The AUC for the normal plan was 0.9911, suggesting that the model has robust evaluative capabilities for both normal and error plans. The AUC for gantry errors was a perfect 1, indicating the model's exceptional ability in evaluating gantry error types, akin to the capacity to distinguish normal plans from error plans through the GPR.

**FIGURE 8:**
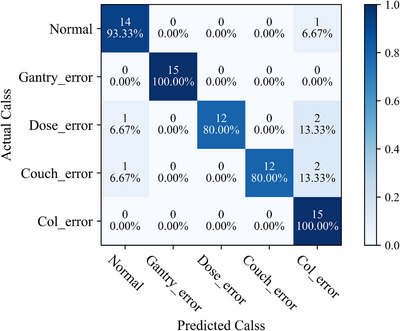
The confusion matrix for multi‐class prediction of the testing dataset in the model.

The confusion matrix of the multi‐classification validation dataset is represented in Figure 8. Within this, 30 instances of normal plan and gantry error plan were successfully classified, although there were some false positives encountered in the classification of the normal plan. The ability to classify dose error plans was subpar, mirroring the subsequent results of the GPR evaluation. The various numeric values associated with the predictive model are displayed in Table 4. The accuracy stands at 0.907, precision at 0.925, recall at 0.907, and the F1 score at 0.908 for IMRT and VMAT plans. Additionally, for IMRT‐only plans, the accuracy stands at 0.881, precision at 0.883, recall at 0.841, and the F1 score at 0.839; For VMAT‐only plans, the accuracy stands at 0.941, precision at 0.955, recall at 0.941, and the F1 score at 0.943. These figures indicate that the overall performance of the model is commendable, with a high degree of accuracy in distinguishing between multiple types of errors.

**TABLE 4:** The performance indicators of the CNN model.

	Techniques for dataset	Accuracy	Precision	Recall	F1 score
CNN	IMRT & VMAT	0.907	0.925	0.907	0.908
	IMRT	0.881	0.883	0.841	0.839
	VMAT	0.941	0.955	0.941	0.943

Abbreviations: IMRT, intensity‐modulated radiation therapy; VMAT, volumetric modulated arc therapy.

Given that the GPR lacks the capacity to assess multi‐classification errors, to facilitate a comparison with the present model, we transformed the multi‐classification outcomes into binary classification results, corresponding to different error types, and compared these against various GPR thresholds. Table 5 illustrates that the CNN model utilized in this study exhibits superior classification capabilities in contrast to the GPR method. Of these, the most successful classification result using the GPR method was the distinction between gantry error plans and normal plans, with respective accuracies of 0.625, 0.575, and 0.667. Conversely, the least successful result was the differentiation between dose error plans and normal plans, which had accuracies of 0.500, 0.500, and 0.500 respectively, suggesting that the GPR method was incapable of judging all error plans. On the other hand, the CNN model employed in this study accomplished an accuracy of 0.960, a precision of 0.875, a recall of 0.933, and an F1 score of 0.903 in predicting dose error plans and other types. Additionally, commendable outcomes were obtained for other classifications as well.

**TABLE 5:** The evaluation results of the CNN model and different GPR thresholds.

Test data types	Evaluation method	Accuracy	Precision	Recall	F1 score
Normal vs. others	CNN model	0.960	0.875	0.933	0.903
	3%/3 mm	0.267	0.214	1.000	0.353
	3%/2 mm	0.260	0.213	1.000	0.351
	2%/2 mm	0.333	0.231	1.000	0.375
Gantry vs. others	CNN model	1.000^b^	1.000	1.000	1.000
Gantry vs. normal	3%/3 mm	0.625	1.000	0.250	0.400
	3%/2 mm	0.575^b^	1.000	0.150	0.261
	2%/2 mm	0.667	1.000	0.333	0.500
Dose vs. others	CNN model	0.960	1.000	0.800	0.889
Dose vs. normal	3%/3 mm	0.500^a^	0.000	0.000	0.000
	3%/2 mm	0.500^a^	0.000	0.000	0.000
	2%/2 mm	0.500^a^	0.000	0.000	0.000
Couch vs. others	CNN model	0.960	1.000	0.800	0.889
Couch vs. normal	3%/3 mm	0.550	1.000	0.100	0.182
	3%/2 mm	0.559	1.000	0.117	0.210
	2%/2 mm	0.633	1.000	0.267	0.421
Collimator vs. others	CNN model	0.933	0.750	1.000	0.857
Collimator vs. normal	3%/3 mm	0.541	1.000	0.083	0.154
	3%/2 mm	0.517	1.000	0.333	0.065
	2%/2 mm	0.533	1.000	0.067	0.125

^a^ Representing accuracy = 0.5, the type of test data cannot be determined accurately.

^b^ Representing accuracy = 1, the type of test data can be accurately determined.

### 3.3 Model prediction results under the different accelerators

To investigate the performance of the prediction model on different accelerators, we randomly predicted 50 radiation therapy plans on Varian VitalBeam for 15 patients. The results shown in Table 6 indicate that the model performance is similar when compared to the Trilogy accelerator, indicating the potential for improvement in robustness. The accuracy stands at 0.900, precision at 0.918, recall at 0.900, and the F1 score at 0.898 for IMRT and VMAT plans. Meanwhile, the results were still superior to those produced by the GPR method. Specifically, Table 7 show that the prediction result on the gantry error was the same as that obtained with the GPR method, but the ability to distinguish dose error was still much stronger using our model than with the GPR method.

**TABLE 6:** The performance indicators of the CNN model on VitalBeam linear accelerator.

	Techniques for dataset	Accuracy	Precision	Recall	F1 score
CNN	IMRT & VMAT	0.900	0.918	0.900	0.898
	IMRT	0.900	0.910	0.880	0.876
	VMAT	0.900	0.929	0.900	0.896

Abbreviations: IMRT, intensity‐modulated radiation therapy; VMAT, volumetric modulated arc therapy.

**TABLE 7:** The evaluation results of the CNN model and different GPR thresholds on VitalBeam linear accelerator.

Test data types	Evaluation method	Accuracy	Precision	Recall	F1 score
Normal vs. others	CNN model	0.980	0.909	1.000	0.952
	3%/3 mm	0.360	0.238	1.000	0.385
	3%/2 mm	0.380	0.244	1.000	0.392
	2%/2 mm	0.380	0.244	1.000	0.392
Gantry vs. others	CNN model	0.980	0.909	1.000	0.952
Gantry vs. normal	3%/3 mm	0.700	1.000	0.400	0.571
	3%/2 mm	0.750	1.000	0.500	0.667
	2%/2 mm	0.750	1.000	0.500	0.667
Dose vs. others	CNN model	0.960	1.000	0.800	0.889
Dose vs. normal	3%/3 mm	0.500^a^	0.000	0.000	0.000
	3%/2 mm	0.500^a^	0.000	0.000	0.000
	2%/2 mm	0.500^a^	0.000	0.000	0.000
Couch vs. others	CNN model	0.940	1.000	0.700	0.824
Couch vs. normal	3%/3 mm	0.600	1.000	0.200	0.333
	3%/2 mm	0.600	1.000	0.200	0.333
	2%/2 mm	0.650	1.000	0.300	0.462
Collimator vs. others	CNN model	0.940	0.769	1.000	0.870
Collimator vs. normal	3%/3 mm	0.600	1.000	0.200	0.333
	3%/2 mm	0.550	1.000	0.100	0.182
	2%/2 mm	0.550	1.000	0.100	0.182

^a^ Representing accuracy = 0.5, the type of test data cannot be determined accurately.

**FIGURE 9:**
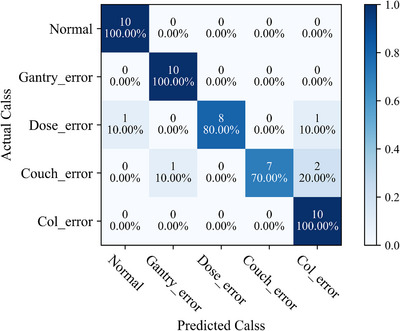
The confusion matrix for multi‐class prediction of the verification dataset in the model.

The confusion matrix of the multi‐classification for verification dataset in Vitalbeam accelerators is represented in Figure 9. Within this, 20 instances of gantry error and dose error were successfully classified. In addition, the model's ability to classify dose error plans is similar to the predictions of the test set.

## 4 DISCUSSION

In this study, we developed a CNN model capable of predicting error types arising during the validation of the radiotherapy plan, thus demonstrating the practicality of applying deep learning for QA in patient radiation therapy plans. The findings suggest that multi‐classification CNN models are capable of detecting and identifying a diverse array of errors in radiation therapy plans, including gantry error, dose error, collimator error, and treatment couch error, with greater sensitivity compared to the currently utilized GPR method. The novelty of this study resides in the application of internationally recognized QA tolerance limits for linear accelerator mechanical precision to formulate error types and the classification prediction of different treatment sites and modulation techniques employing 3D dose measurement. The model yielded satisfactory results on several types of accelerators, showcasing its robustness and providing a novel avenue for patient‐specific QA in clinical radiotherapy.

Existing research has demonstrated the potential for utilizing deep learning in predicting the GPR for QA in radiation therapy plan dose verification. However, few studies have delved into error detection by analyzing the measured dose distribution.^29^ Some studies have proposed the use of machine learning‐based methods for analyzing individual static beam IMRT or VMAT plans. Potter et al.,^30^ for example, analyzed errors such as MLC movement, MLC transmission factor, dose, and radiation source size using machine learning. This was achieved by extracting features like distance to agreement (DTA) and DD based on a 2D flat detector array method. Wolfs et al.^31^proposed an error recognition model utilizing a CNN model for EPID dose measurement for lung cancer patients, simulating both mechanical and patient‐induced errors such as patient setup errors and tumor regression.

In contrast, our study applied a CNN model and 3D dose measurement to execute multi‐ classification for error detection in radiation therapy plans for different sites. Therefore, it is challenging to compare the accuracy performance of our model directly with those from prior studies. However, our model appears to be more sensitive to gantry errors compared to previous studies that analyzed two‐dimensional dose maps using 2D diode arrays or EPID. This increased sensitivity arises from the positional relationship between the radiation source direction and the detector device. In previous research using EPID, the detector position was always perpendicular to the beam for either static beam IMRT or VMAT plans, making it impossible to detect rotational errors in the gantry. Moreover, the sensitivity to gantry errors in VMAT plans measured using a 2D diode array is weak due to the different angles of radiation transmission and the cumulative dose measurement, complicating error detection.^32^

In this study, our model showed the highest sensitivity to gantry errors and delivered impressive results for normal plans, couch, collimator, and dose errors. In contrast, the GPR method only achieved results comparable to our model for gantry error type. This discrepancy arises because the GPR method is primarily constructed to compensate for limitations associated with comparing only the DD or the distance difference when dramatic changes occur in dose regions or areas of low dose. Unfortunately, this leads to a somewhat biased comparison of the radiation therapy plan's dose distribution. Therefore, when only a dose error is present, the detection sensitivity of such errors is considerably reduced using the DTA method. This method seeks to locate the closest point with a similar dose to the measurement point within the TPS for comparison. Regrettably, the GPR method can only provide binary classification results, determining whether the plan passes or not, and cannot accurately classify types of error. In contrast, our model can accomplish this. Some recent studies directly predict plan verification success or failure based on the fluence of the TPS dose. However, such methods cannot offer feedback on accelerator errors during implementation. The dose analysis method reliant on measurements in our study can address this issue by optimizing the model structure and generating error data used in the training process.

Despite the significant advantages, our method comes with some inherent limitations. Primarily, our approach is based on supervised learning and can only detect errors that have been incorporated during the training process. As a result, it is necessary to integrate several types of error into the model to enhance its adaptability and scope of application. For the realization of an optimal deep learning‐based error detection system, it is imperative to develop a model capable of classifying errors without the need to specify error types. To address this challenge, an unsupervised or semi‐supervised learning deep learning method might serve as a more efficient alternative to the GPR method. Furthermore, the better approach is to collect and train data from multiple centers and large models for each tumor and technology. This is also the direction we will continue to research in the future. However, based on the existing center datasets, our results demonstrate that the model already has good error prediction capabilities to complement the GPR method. Additionally, for VMAT plans, we were unable to simulate errors in the gantry rotation.

However, our study boasts significant strengths, including the utilization of IMRT and VMAT plans from varied regions for error classification. This allows for a more versatile application of the model across diverse radiation treatment plans. Moreover, our model demonstrated commendable overall plan error classification performance, providing a more clinically accurate composite dose distribution analysis than prior studies that classified errors by partitioning multiple fields of an individual plan. The methodology mirrors real clinical situations more closely, which marks it as a promising tool for future clinical applications. Moreover, by validating the results across different accelerators, this study has confirmed the practical applicability of this predictive model in clinical settings, thereby compensating for the limitations of the GPR method.

## 5 CONCLUSION

This study established a CNN based predictive model capable of multi‐class error classification. This model employs 3D dose measurements of radiation treatment plans across different treatment sites and techniques to predict and classify error types that exceed the set tolerance guidelines during radiation therapy validation procedures. We have evaluated and verified the model's performance for five distinct classes of errors (normal plans, gantry rotation errors, dose errors, treatment couch errors, and collimator errors) across various accelerator types. The findings indicate that our proposed model outperforms the GPR method in detecting diverse types of errors. This method, in synergy with the GPR method, may serve as a novel QA approach, effectively addressing the limitations of the GPR method.

## AUTHOR CONTRIBUTIONS

Creation of the new deep learning model, model testing, performing measurements/experiments design of the experiments, and writing of the manuscript: Dr. Shupeng Liu. Participation in the concept of the project and participation in the design of experiments: Dr. Jianhui Ma. Participation in the design of the model and critical revision of the manuscript: Fan Tang. Acquisition of the clinical data: Yuqi Liang, Yanning Li, Zihao Li, and Tingting Wang. Funding acquisition and approval of the version of the manuscript: Prof. Meijuan Zhou.

## CONFLICT OF INTEREST STATEMENT

The authors declare no conflicts of interest.
